# Effect of Chaihu-jia-Longgu-Muli decoction on withdrawal symptoms in rats with methamphetamine-induced conditioned place preference

**DOI:** 10.1042/BSR20211376

**Published:** 2021-08-20

**Authors:** Zifa Li, Yuchen Qi, Kun Liu, Yiming Cao, Hao Zhang, Chunhong Song, Hualiang Deng

**Affiliations:** 1 Behavioural Phenotyping Core Facility, Shandong University of Traditional Chinese Medicine, Jinan 250355, China; 2 Chinese Medicine Neuro-Psycho Pharmacology Laboratory (CMNPPL), Shandong University of Traditional Chinese Medicine, Jinan 250355, China; 3 No. 2 Department of Encephalopathy, Affiliated Hospital of Shandong University of Traditional Chinese Medicine, Jinan 250011, China; 4 School of Traditional Chinese Medicine, Shandong University of Traditional Chinese Medicine, Jinan 250355, China

**Keywords:** 5-hydroxytryptamine (5-HT), Chaihu-jia-Longgu-Muli-decoction (CLMD), Dopamine (DA), Endorphins (β-EP), Interleukins, methamphetamine

## Abstract

Traditional Chinese medicine detoxification prescription Chaihu-jia-Longgu-Muli decoction (CLMD) relieves depressive symptoms in patients withdrawing from methamphetamine. In the present study, we assessed the effects of CLMD on methamphetamine withdrawal in rats. A methamphetamine-intoxicated rat model was established. Rats were randomly divided into the control, model, high-dosage, medium-dosage, and low-dosage groups, receiving high, medium, and low doses of CLMD, respectively. Weekly body weight measurements revealed that rats treated with methamphetamine had the lowest body weight. The conditioned place preference (CPP) experiment revealed that methamphetamine-intoxicated rats stayed significantly longer in the drug-paired chamber than the control rats. However, after administering high-dosage CLMD, the amount of time the rats spent in the drug-paired chamber was significantly less than that of the model rats. Our open-field test revealed that the model group had lower crossing and rearing scores than the control group. Additionally, rats that received CLMD treatment exhibited higher crossing and rearing scores than the model rats. Striatal dopamine (DA), 5-hydroxytryptamine (5-HT), and endorphins (β-EP) and serum interleukin (IL)-1α and IL-2 concentrations were estimated. Rats in the model group had lower striatal DA, 5-HT, and β-EP and higher serum IL-1α and IL-2 concentrations than those in the control group. High-dosage CLMD administration significantly changed the concentrations of these molecules, such that they approached normal concentrations. In general, CLMD could prevent the development of methamphetamine-induced withdrawal symptoms in rats by increasing the DA, 5-HT, and β-EP and lowering the IL-1α and IL-2 concentrations.

## Introduction

Methamphetamine use is associated with an array of symptoms, such as mental excitation, loss of appetite, insomnia [1], and inclination toward social violence. Neurotoxicity in multiple neurotransmitter systems arises as a result of methamphetamine consumption [2,3]. By altering synaptic plasticity in the brain, methamphetamine use can result in adverse effects such as dependence, withdrawal syndrome, and cravings [4,5]. Once a cut-off concentration is reached, withdrawal symptoms manifest [6]. Anxiety and depression are two common symptoms of methamphetamine withdrawal and may be associated with cravings and drug dependency [7].

Methamphetamine stimulates the release of several neurotransmitters such as dopamine (DA), 5-hydroxytryptamine (5-HT), and endorphins (β-EP), which are associated with emotions [8–10]. Methamphetamine directly acts on the dopaminergic neurons and competes with released DA for access to DA transporters [11]. It then destroys the DA storage vesicles and facilitates DA antiport using the transporters excreted by DA [11]. In addition, methamphetamine can lead to neuronal death in different cerebral areas, such as the striatum. Methamphetamine exposure can damage the dopaminergic neurons in the substantia nigra, leading to lower DA concentration within the striatum [12,13]. The chemical structure of methamphetamine is similar to that of catecholamine-related neurotransmitters; therefore, it can enter into the neuronal ends through 5-HT transporters to replace 5-HT in vesicles and cells. During this process, significant levels of 5-HT are consumed, leading to damage to the neuronal ends that contain 5-HT [14]. The neurotoxicity associated with the intake of methamphetamine causes damage to the dopaminergic and serotoninergic ends of the neurons as well as to the nigrostriatal pathway [15]. β-EP produces reward effects by combining with the μ receptor, leading to feelings of satisfaction and euphoria [16]. The μ receptors are widely distributed throughout the central nervous system and are broadly recognized as opiate receptors associated with addiction [17].

The immune system also plays an important role in the pathogenesis of neuropsychiatric disorders, including cognitive decline, anxiety, mood changes, and depressive states, as well as increased attention, decreased fatigue, and the rush of euphoria [18–20], which are associated with methamphetamine use. Inflammatory biomarkers, especially interleukin (IL)-1 (IL-1), are increased by methamphetamine use and are involved in methamphetamine-induced neurodegeneration [21–23]. IL-2 (a potent T-cell growth factor) levels have been found to be significantly higher in hypothalamic samples taken from methamphetamine-exposed mice [24].

To date, most studies have focused on exploring the mechanisms of neuropsychiatric disorders and immune dysregulation related to methamphetamine use and have not clarified the behavioral changes leading to its abuse or aided in the development of rehabilitation medicines with few side effects. Chaihu-jia-Longgu-Muli decoction (CLMD) is a detoxifying formulation containing herbal medicine based on the basic theory of traditional Chinese medicine. CLMD has been used as a remedy for many years with very few side effects and has been frequently used clinically for the treatment of neuropsychiatric disorders [25]. In addition to having few associated toxic effects, CLMD has significant beneficial effects on methamphetamine-induced depressive symptoms, which occur after withdrawal [26]. There is a large body of research indicating that this formulation and its derivatives are effective in reducing intimal thickening of the carotid artery in animal models. In mice, not only has antidepressant activity and reduction in chronic mild stress-induced apoptosis in the hippocampus been observed but also the treatment of insomnia and improvement in sleep quality have been noted [25,27–29]. However, little is known about the effect of CLMD on behavioral responses to amphetamine withdrawal symptoms. Based on the clinical effects of CLMD seen in methamphetamine-addicted people after withdrawal [26,30], the present study was conducted to verify the effects of CLMD on the behavior of rats withdrawing from methamphetamine in an attempt to explore the signaling pathways involved. We further aimed to estimate the extent of recovery from neuropsychiatric disorders and immune dysregulation resulting from CLMD treatment, especially striatal DA, 5-HT, and β-EP and serum IL-1α and IL-2 concentrations, and explored the functional mechanisms of CLMD.

## Materials and methods

### Animals

Sixty SPF male Sprague–Dawley rats weighing 170–190 g were provided by the Beijing Vital River Laboratory Animal Technology Co., Ltd., Beijing, China (laboratory animal production license no. SCXK [BJ]2016-0006). All animal experiments took place at the Behavioral Phenotyping Core Facility, Shandong University of Traditional Chinese Medicine, and the animals were adapted to the following experimental conditions for 1 week: temperature: 21 ± 1°C; humidity: 40 ± 5%; and a 12-h light/dark cycle (light on at 20:00 and off at 8:00). The animals were fed a standard diet and filtered water *ad libitum*. The research plan and experimental procedures followed a protocol approved by the Animal Use and Care Committee of Shandong University of Traditional Chinese Medicine, Jinan, China (ethics approval reference no. SDUTCM2018-072), and were conducted according to the Guide for the Care and Use of Laboratory Animals.

### Preparation of drugs and reagents

Methamphetamine, which was provided by the Detoxification Surveillance and Treatment Center of China (Shandong branch), was dissolved in saline immediately before intramuscular injection at a dose of 2 mg/kg. All other chemicals used in the present study were purchased from Sinopharm Chemical Reagent Co., Ltd (Shanghai, China).

CLMD was prepared according to the following steps. First, Radix bupleuri (36 g), ginseng (15 g), fossil fragments (12 g), oyster (12 g), Radix scutellariae (12 g), ochre (12 g), Cassia twig (12 g), Tuckahoe (12 g), pinellia ternata (9 g), *Rheum officinale* (9 g), ginger (12 g), and Chinese dates (41 g) were purchased from the Shandong Pharmaceutical Company (Jinan, China). The ingredients were boiled twice in a volume of water ten-times that of the ingredients. Two batches of filtered soup were mixed using filter paper (Nanjing Wanqing Chemical Glassware Instrument Co., Ltd.). The filtered soup was then dried by distillation and converted into a freeze-dried powder extract, which was stored at −20°C.

### Establishment of the model, drug administration, and animal groups

**Figure 1:**
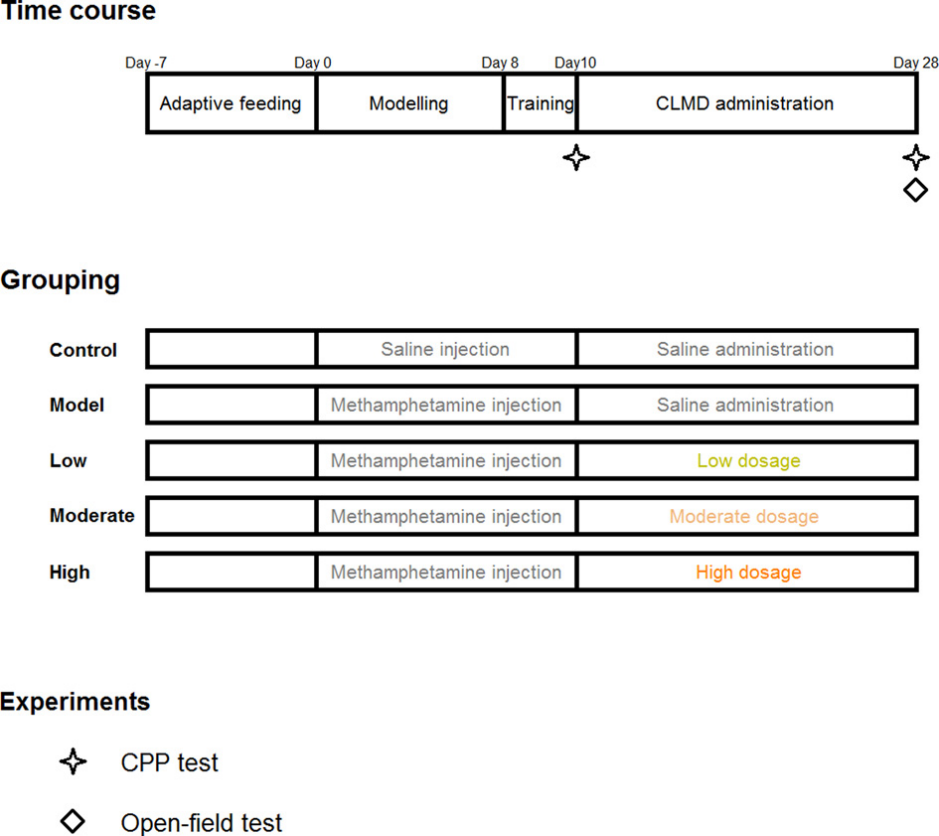
Experimental design Schedule of the experimental design including time course, grouping, and timing of behavioral experiments.

Fifty-five pre-qualified rats were selected and assigned randomly into five groups: the control, model, high-dosage (20 mg/kg), moderate-dosage (10 mg/kg), and low-dosage (5 mg/kg) groups, with 11 rats in each group. The dried powder extract was weighed for each group and diluted in appropriate volume of water. The absolute volume of the CLMD liquor administered to each rat was calculated according to their individual body weights so that the relative administration volume for all the rats was fixed at 0.5 ml/100 g of weight. Dosages administered in the high-, moderate-, and low-dosage groups were equivalent to 10-, 5-, and 2.5-times the clinical dosages, respectively. All groups, except the control group, received intramuscular injections of 2 mg/kg methamphetamine daily for 10 days to establish the methamphetamine-intoxicated rat model for the conditioned place preference (CPP) test (Figure 1). The control group was administered the same dosage of saline by intramuscular injection. After injection, the corresponding dosages of CLMD were administered by gavage for 18 consecutive days (Figure 1). The rats’ body weights were measured once every week, and the administrations were adjusted accordingly.

### CPP experiment

Before initiating the experiment, the rats were placed inside a CPP apparatus for adaptive feeding for 7 days. The CPP apparatus consisted of two equal-sized compartments (30 × 30 × 43 cm), one with a white box and the other with a black box joined by a wall with a sliding door. The ‘non-drug-paired’ chamber was black, while the ‘drug-paired’ chamber was white. The length of time the rats actively stayed within each of the two chambers was recorded, and those that actively stayed longer in the ‘drug-paired’ chamber than in the ‘non-drug-paired’ chamber were rejected.

After 8 days of establishing the model (saline injections for the control group and methamphetamine injections for the model and CLMD treatment groups), the rats were placed inside the ‘drug-paired’ chamber and received intramuscular injections of methamphetamine in the morning (a clapboard was placed between the chambers so that the rats could only stay in the ‘drug-paired’ chamber). The rats were taken out after 30 min. At the same time, the control experiment was conducted. The rats in the control group were also placed inside the ‘drug-paired’ chamber after intramuscular injection of the same dosage of saline and then taken out after 30 min. The training phase continued for 2 days, and the CPP test was conducted on the tenth day (Figure 1).

No drugs were administered during the CPP test. The rats were placed inside the passage close to the two chambers, and the clapboard was lifted to allow them to freely move between the two chambers. The test time was 15 min. The length of time that the rats stayed within each chamber was recorded. The CPP test was re-conducted 24 h after 18 days of CLMD treatment (Figure 1), with a test time of 15 min.

### Open-field test

The behavior of the rats was observed by the open-field test. The field test chamber had a dimension of 50 cm × 50 cm × 50 cm, with an open top, black baffles on the sides and bottom, and a Sudoku design at the bottom as a test base. One day before the test, the rats were placed into the field test chamber for 10 min to adapt them to the experimental environment. This adaptation was expected to decrease the influence of the strange environment on the activities of the rats. Upon initiating the experiment, the rats were lightly placed on the central grid of the field test chamber, and their activity status was recorded for 5 min automatically using a video analysis system. The number of grids crossed, total length of movement, and number of times they stood erect were recorded. The horizontal and vertical scores of the rats were calculated. The horizontal score was the total number of grids crossed at the bottom, while the vertical score was the number of times the rat stood erect, including raising its forepaws into the air or using the wall for support [31].

### Estimation of striatal DA, 5-HT, and β-EP concentrations serum and IL-1α and IL-2 concentrations

After the behavioral tests, the rats were anesthetized with pentobarbital sodium and killed by neck breaking. Blood samples were collected and centrifuged for 15 min at 3000×***g*** to obtain the serum. The rats were then killed to obtain 100 mg of striatal tissue, which was put into Eppendorf Safe-Lock tubes after washing the blood stain with phosphate buffered saline (PBS), followed by the addition of 1 ml of PBS. The mixture was then homogenized with a tissue grinder and placed at −20°C overnight. After two freeze-thaw cycles to damage the cell membranes, the homogenate was centrifuged at 5000×***g*** for 5 min at 4°C to obtain the supernatant. Striatal DA, 5-HT, and β-EP and serum IL-1α and IL-2 concentrations were estimated in strict accordance with the instructions of the following ELISA kits: DA ELISA (Wuhan Huamei Biotech Co., Ltd.; batch no. Y06015074), 5-HT ELISA (Wuhan Huamei Biotech Co., Ltd.; batch no. C0150040107), β-EP ELISA (Wuhan Huamei Biotech Co., Ltd.; batch no. C0150050108), IL-2 ELISA (Wuhan Huamei Biotech Co., Ltd.; batch no. C23015075), and IL-1α ELISA (Wuhan Huamei Biotech Co., Ltd.; batch no. Y02015076).

### Statistical analyses

All data are represented as mean ± SD and were analyzed using GraphPad Prism version 6.0.1 (GraphPad Software, Inc., San Diego, California, U.S.A.). The results were analyzed using unpaired *t* tests or two-way analysis of variance (ANOVA). Post hoc tests were performed following ANOVA where appropriate. A *P*-value <0.05 was considered statistically significant.

## Results

### Changes in body weight

**Figure 2:**
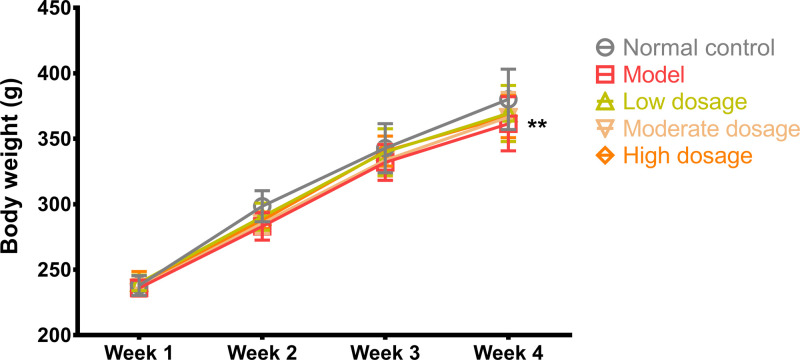
Body weight Body weight of the rats in the control, model, CLMD low-dosage, CLMD moderate-dosage, and CLMD high-dosage groups. *n*=12. **P<0.01 vs. control group.

Body weights of the rats before and after the experiment for each group were not significantly different. However, the body weights of the rats in the model group were significantly different compared with those of the rats in the control group (361.4 ± 20.69 g vs. 380.1 ± 23.03 g, *P*<0.01, q = 4.695, DF = 212). The body weights of the rats in different treatment groups increased compared with those of the rats in the model group, but the difference was not significant. The results are shown in Figure 2.

### CPP

**Figure 3:**
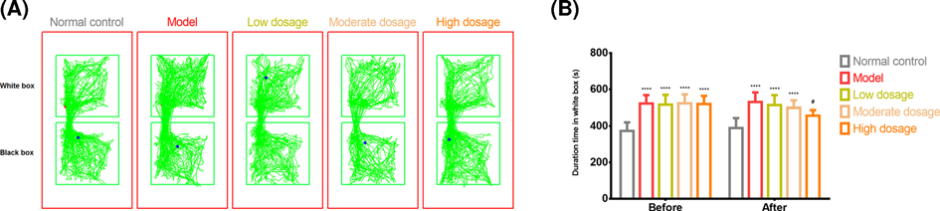
CPP test CPP test results from the control, model, CLMD low-dosage, CLMD moderate-dosage, and CLMD high-dosage groups. (**A**) The trajectory for the five groups. (**B**) Length of time spent within the white box for the five groups. ****P<0.0001 vs. control group; ^#^*P*<0.05 vs. model group.

The ‘non-drug-paired’ chamber was black, while the ‘drug-paired’ chamber was white. We recorded the duration for which the rats stayed within the white box (Figure 3A). The rats in the model group stayed significantly longer in the drug-paired chamber before the experiment than those in the control group (373.7 ± 47.0 s vs. 522.0 ± 46.6 s, *P*<0.0001, t = 7.232, DF = 100, Figure 3B). After CLMD administration, the difference in the duration for which the rats in the model group and those in the high-dosage group stayed in the drug-paired chamber was statistically significant (530.6 ± 52.7 s vs. 457.8 ± 30.0 s, *P*<0.05, t = 3.551, DF = 100, Figure 3B). However, there was no significant difference in the duration for which the rats stayed in the white box after CLMD administration between the model and moderate-dosage groups or between the model and low-dosage groups (Figure 3B).

### Open-field test

**Figure 4:**
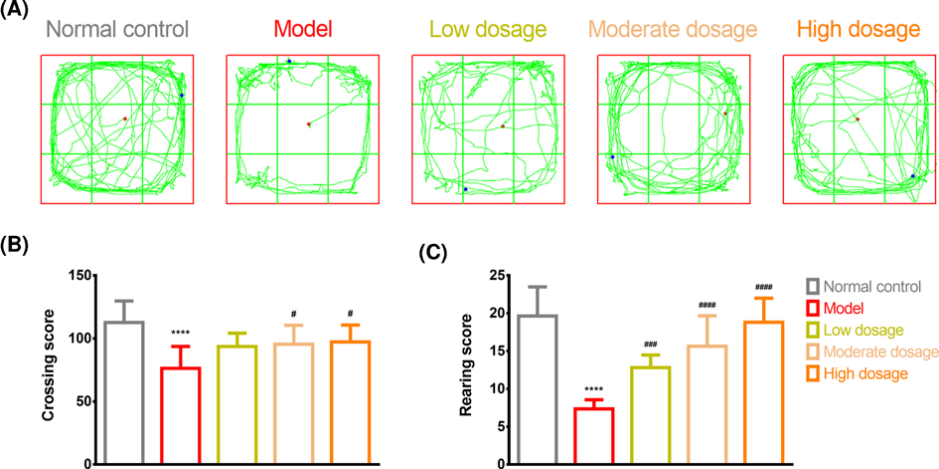
Open-field test Open-field test results for the control, model, CLMD low-dosage, CLMD moderate-dosage, and CLMD high-dosage groups. (**A**) Trajectory for the five groups. (**B**) Crossing score for the five groups. (**C**) Rearing score for the five groups. ****P<0.0001 vs. control group. ^#^*P*<0.05 vs. model group; ^###^*P*<0.001 vs. model group; ^####^*P*<0.0001 vs. model group.

Figure 4A traces the movement of the rats in the open-field test for the different groups. The rats in the model group had lower crossing (112.73 ± 17.03 vs. 76.36 ± 17.34, *P*<0.0001, q = 8.101, DF = 50, Figure 4B) and rearing (19.64 ± 3.85 vs. 7.36 ± 1.21, *P*<0.0001, q = 13.51, DF = 50, Figure 4C) scores, as demonstrated by fewer number of grids crossed, reduced activity, and fewer number of times standing erect or using the wall for support, than those in the control group. The moderate-dosage (95.45 ± 15.07) and high-dosage (97.27 ± 13.40) groups exhibited higher crossing scores than the model group (76.36 ± 17.34, *P*<0.05, q = 4.657, DF = 50, Figure 4B). The low-dosage (12.82 ± 1.66), moderate-dosage (15.64 ± 4.03), and high-dosage (18.82 ± 3.19) groups had higher scores than the model group (7.36 ± 1.21, *P*<0.0001, Figure 4C) (low-dosage vs. model group: q = 6.006, DF = 50; moderate-dosage vs. model group: q = 9.108, DF = 50; and high-dosage vs. model group: q = 12.61, DF = 50). Notably, higher dosages of CLMD led to higher crossing and rearing scores (Figure 4B,C).

### Detection of striatal DA, 5-HT, and β-EP and serum IL-1 and IL-2 concentrations

**Figure 5:**
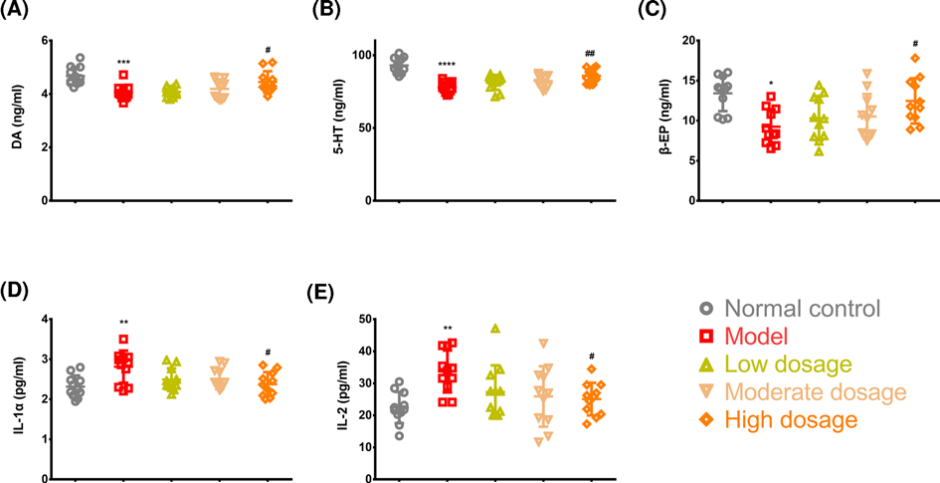
Key molecule levels in the striatum and serum Detection of striatal DA (**A**), 5-HT (**B**), and β-EP (**C**) and serum IL-1 (**D**) and IL-2 (**E**) concentrations. *P<0.05 vs. control group; **P<0.01 vs. control group; ***P<0.001 vs. control group; ****P<0.0001 vs. control group; ^#^*P*<0.05 vs. model group; ^##^*P*<0.01 vs. model group.

Striatal DA, 5-HT, and β-EP concentrations were significantly lower in the rats in the model group than in those in the control group (DA, *P*<0.001, q = 6.393, DF = 50; 5-HT, *P*<0.0001, q = 10.58, DF = 50; and β-EP, *P*<0.05, q = 5.38, DF = 50) (Figure 5A–C). There were significant differences in DA, 5-HT, and β-EP concentrations between the model and high-dosage groups (DA, *P*<0.05, q = 4.096, DF = 50; 5-HT, *P*<0.01, q = 5.649, DF = 50; and β-EP, *P*<0.05, q = 4.156, DF = 50) (Figure 5A–C). The model group exhibited higher serum IL-1 and IL-2 concentrations than the control group (both *P*<0.01). Additionally, high-dosage CLMD significantly reduced serum IL-1 and IL-2 concentrations more in the high-dosage group than in the model group (both *P*<0.05, q = 4.703, q = 4.039, DF = 50).

## Discussion

### Methamphetamine-induced CPP model identification

The CPP test is a classical experimental model that is commonly used in disciplinary research related to rehabilitation, learning memory, behavior functions, and new drug development or drug screening to evaluate psychological dependence on various drugs [32]. Typical drug abuse-induced behavioral changes are normally mediated through the midbrain mesolimbic DA system, and even drug abuse can lead to different behavioral changes [33]. In this study, the conditioned rats demonstrated a preference for the drug-free environment that had previously been paired with methamphetamine. When methamphetamine administration was repeatedly associated with specific surroundings, these served as a cue and induced positive subjective feelings even in the absence of methamphetamine (Figure 3). Therefore, we successfully developed an animal model by administering rats with methamphetamine and observing their levels of anxiety, depression, and body weight after drug withdrawal (Figure 2). Our findings showed that high-dosage CLMD could prevent the formation of methamphetamine-induced CPP. A lower intensity of addiction was accompanied by lower craving for methamphetamine, which aided in the amelioration of symptoms from the already formed CPP, as shown in the CPP tests (Figure 3). In the open-field tests, the rats in the model group exhibited reduced activity and fewer episodes of standing or using the wall to support themselves to stand erect, suggesting that during methamphetamine withdrawal, the rats developed generalized anxiety and depressive symptoms. Therefore, CLMD treatment could effectively relieve withdrawal symptoms such as anxiety and depression (Figure 4).

### Changes in the nervous system in the methamphetamine-induced CPP model

The mechanisms of drug addiction have already been well established. This involves the disruption of the natural well-balanced learning and memory system related to reward [34]. At an early stage of intake, the whole reward system is stimulated by methamphetamine, thereby activating the natural reward system. However, after long-term intake, DA must be reduced gradually so that the function of the whole reward system is not compromised as a result of the lower concentration of DA and fewer number of DA receptors [35]. Biochemical and neuroimaging research on patients using methamphetamines have also shown lower concentrations of DA and its transport protein and the activation of microglial cells in the striatum and other cerebral areas [36]. These findings are consistent with our results (Figure 5A).

Additionally, the neurotoxicity of methamphetamine leads to damage to the dopaminergic and serotoninergic neurons in the nigrostriatal pathway [15]. Therefore, we observed that after 24 h of withdrawal from methamphetamine, striatal DA and 5-HT concentration was significantly lower in the rats in the model group, suggesting that withdrawal symptoms experienced by these rats were associated with lower DA and 5-HT concentrations (Figure 5A,B). Fortunately, through CLMD treatment, we also identified that striatal DA and 5-HT concentrations were up-regulated in the rats in the high-dosage group, exerting certain inhibitory effects on the withdrawal symptoms that might be associated with the increased striatal DA and 5-HT concentrations (Figure 5A,B).

During the course of addiction, β-EP concentration increases due to the stimulation of methamphetamine [8–10]. This is a similar effect to what occurs with an influx of a large number of exogenous opiates into the body. During the withdrawal phase, due to the suspension of methamphetamine stimulation, endogenous β-EP concentration is lower, causing peripheral and central withdrawal symptoms [37], which were also exhibited in our model (Figure 5C). CLMD treatment up-regulated striatal β-EP concentration, which could also contribute to ameliorating withdrawal symptoms.

### Changes in serum IL concentrations in the methamphetamine-induced CPP model

Studies have shown that methamphetamine toxicity-induced neuronal injury is mediated through the activation of the microglial cell response and tumor necrosis factor system [38,39]. Some drugs that inhibit the immune response can reduce drug dependence by partly reducing the activation of methamphetamine-dependent microglial cells [40]. Another study reported that microglial activation leads to an inflammatory response in the neurons and that by inhibiting microglial activation, the expression of inflammatory factors is reduced in a methamphetamine poisoning rat model [41]. Our finding is consistent with these findings. In our results, higher concentrations of IL-1α and IL-2, which are two important inflammatory factors, were observed in the methamphetamine-induced CPP model than in the control rats (Figure 5D,E), suggesting that methamphetamine poisoning was associated with elevated IL-1α and IL-2 concentrations. CLMD treatment reduced IL-1α and IL-2 concentrations in the methamphetamine-intoxicated rats (Figure 5D,E); however, the mechanism by which CLMD exerts its effects on IL-1α and IL-2 concentrations was not clarified.

## Conclusions

By relieving or treating anxiety, depressive symptoms, and somnipathy, CLMD inhibited the methamphetamine-induced formation of CPP, reduced the intensity of the addiction, weakened methamphetamine craving, and resulted in relief from the effects of previously established CPP.

## Data Availability

The datasets analyzed during the study are available from the corresponding author on reasonable request.

## Abbreviations

ANOVA: analysis of variance
CLMD: Chaihu-jia-Longgu-Muli decoction
CPP: conditioned place preference
DA: dopamine
DF: degree of freedom
IL: interleukin
SPF: specific pathogen free
5-HT: 5-hydroxytryptamine
β-EP: endorphin
